# Occurrence and Impact of Minor Histocompatibility Antigens' Disparities on Outcomes of Hematopoietic Stem Cell Transplantation from HLA-Matched Sibling Donors

**DOI:** 10.1155/2012/257086

**Published:** 2012-11-08

**Authors:** Monika Dzierzak-Mietla, M. Markiewicz, Urszula Siekiera, Sylwia Mizia, Anna Koclega, Patrycja Zielinska, Malgorzata Sobczyk-Kruszelnicka, Slawomira Kyrcz-Krzemien

**Affiliations:** ^1^Department of Hematology and Bone Marrow Transplantation, Medical University of Silesia, Dabrowskiego 25, 40-032 Katowice, Poland; ^2^HLA and Immunogenetics Laboratory, Regional Blood Center, Raciborska 15, 40-074 Katowice, Poland; ^3^Lower Silesian Center for Cellular Transplantation with National Bone Marrow Donor Registry, Grabiszynska 105, 53-439 Wroclaw, Poland

## Abstract

We have examined the alleles of eleven minor histocompatibility antigens (MiHAs) and investigated the occurrence of immunogenic MiHA disparities in 62 recipients of allogeneic hematopoietic cell transplantation (allo-HCT) with myeloablative conditioning performed between 2000 and 2008 and in their HLA-matched sibling donors. Immunogenic MiHA mismatches were detected in 42 donor-recipient pairs: in 29% MiHA was mismatched in HVG direction, in another 29% in GVH direction; bidirectional MiHA disparity was detected in 10% and no MiHA mismatches in 32%. Patients with GVH-directed HY mismatches had lower both overall survival and disease-free survival at 3 years than patients with compatible HY; also higher incidence of both severe acute GvHD and extensive chronic GVHD was observed in patients with GVH-directed HY mismatch. On contrary, GVH-directed mismatches of autosomally encoded MiHAs had no negative effect on overall survival. Results of our study help to understand why posttransplant courses of allo-HCT from siblings may vary despite the complete high-resolution HLA matching of a donor and a recipient.

## 1. Introduction

The allogeneic hematopoietic cell transplantation (allo-HCT) still remains a curative treatment of many severe diseases, especially hematooncological malignancies. The successful donor search is one of the most important factors deciding about the feasibility of transplantation. It starts with search among the patient's siblings as the HLA-matched sibling donor is regarded as the optimal one. The odds ratio for HLA compatibility in siblings is 1 : 4. The probability of having a matched sibling donor by a particular patient is determined by the formula 1 − (0.75)^*n*^, where *n* equals the number of siblings. Despite the improved matching of donor-recipient pairs that was possible after the implementation of high-resolution methods of molecular HLA typing, the better outcomes of transplantations are still limited by high number of complications: graft versus host disease (GVHD), engraftment problems (lack or loss of engraftment), and relapse [1]. The long-term survival after allo-HCT is being estimated in the range of 40–70%. Failures are mainly due to infectious complications and GVHD (30–40% each), organ toxicity of chemotherapy (20%), and relapse (20–30%) [2].

HLA matching remains the most important factor influencing both donor selection and transplantation outcomes. However, research of the human genome revealed that polymorphism of nucleotides in genes that are non-HLA related (e.g., NOD2/CARD15 or genes encoding cytokines: TNF-alpha, IL-10, IL-6, interferon gamma, IL-1, and TGF-beta) may also determine the individual immunological phenotype of donor-recipient pairs, thus influencing GVHD, infections, and overall survival [3]. Minor histocompatibility antigens (MiHAs) belong to immunogenetic non-HLA related factors encoded by polymorphic genes, which may differ between the recipient and the donor and thus they may have impact on transplant outcomes.

The impact of antigens independent from Major Histocompatibility complex on transplantation results was first observed by Counce et al. in 1950s [4]. They explored graft rejection in inbred mice, which had undergone the transplantation of skin cells and neoplasmatic cells. Genes which were not associated with MHC responsible for slower course of rejection were called weak histocompatibility genes [4, 5]. The first hypothesis concerning potential impact of MiHA on the outcome of BMT (bone marrow transplantation) was based on a case of a female recipient (with severe aplastic anemia) who received a transplant from her brother. Graft rejection after BMT was diagnosed and reactivity of cytotoxic T cells isolated from peripheral blood of recipient was directed to antigens present on donor's cells which were not associated with HLA [6].

Minor histocompatibility antigens are polymorphic peptides consisting of 9–12 amino acids. After binding to the antigen recognition site of either class I or class II HLA molecules present on a cell surface MiHAs can be recognized by T-lymphocytes. Thus the occurrence of MiHA depends on the presence of specific HLA antigens, which is called the MHC restriction. MiHAs are encoded by either autosomal chromosomes or by Y-chromosome [7–9]. Disparities of MiHA may result from polymorphism of amino acids, gene deletions [10], or from several intracellular mechanisms [11]. MiHA disparity may originate from a single or several amino-acid substitution in the part of MiHA peptide recognized by TCR (T-cell receptor), like in the case of HY and HA-1. Amino-acid polymorphism may be present in the region of MiHA that binds to HLA molecule, causing different expressions of peptide-HLA complex in the donor than in the recipient. Polymorphism may also pertain proteins responsible for intracellular processing of peptides, what leads to the presence or absence of peptides (e.g., HA-2 or HA-8) on cell's surface [12], or phosphoproteins (e.g., SP-110, MiHA discovered in 2006 by Warren et al.) [13]. 

Most MiHA possess only one immunogenic allele, which is sufficient to induce MiHA immunogenicity [12]. Up to date 18 autosomal and 10 Y-chromosome encoded MiHAs have been identified; those tested in our study are presented in Tables 1 and 2.

There are two patterns of MiHAs' tissue distribution: restricted and broad. Autosomal HA-3, HA-8, and most of MiHAs encoded by Y-chromosome are present in most tissues, including those crucial for GVHD: skin, intestines, and liver [11, 12]. Most of autosomal and 2 MiHAs encoded by Y-chromosome (B8/HY and B52/HY) appear only in hematologic cells including leukemic cells, dendritic cells, NK, and multiple myeloma cells [40]. Thanks to their restricted distribution all of them may be potentially exploited in immunotherapy. The other type of MiHAs' tissue distribution is their appearance on epithelial neoplasmatic cells, for example, HA-1 and ACC-1/ACC-2 [41, 42], although in normal conditions they are restricted only to hematopoietic cells and are not present on epithelial cells.

Detection of MiHA bases most often on genomic typing with PCR-SSP method. The assessment of detected immunogenic disparities is simplified by the online availability of Leiden University Medical Center's dbMinor database [43]. Disparities of immunogenic MiHA alleles between the donor and the recipient may trigger GVHD and HVG reactions, which may lead to graft rejection or to GVH/GVL reaction [44–46]. T-lymphocytes directed against recipient specific MiHAs were detected in patients with GVHD [47]. In the group of 92 recipients of allo-HCT from unrelated donors, a higher incidence of chronic GVHD was observed in those with HY disparity [48]. Many clinical trials confirm that disparities of autosomally encoded MiHAs (like HA-1, HA-2, and HA-8) may increase the incidence of GVHD [15, 17, 22], while others did not confirm such dependence [49]. Female recipients after transplantation from male donors may experience graft failure due to HVG reaction against HY antigens resulting in a worse survival [3]. MiHA present on recipient's neoplasmatic cells (HA-1, HA-2, HA-8, HB-1, and HY) may constitute the target of cytotoxic CD8+ T-lymphocytes crucial for GVL reaction [12, 50], leading to the decrease of relapse rate [51]. Use of cytotoxic T-lymphocytes recognizing selectively only MiHA present on neoplasmatic cells enables the separation of GVL effect from GVHD [52]. Such MiHAs can be used both in vivo for the production of vaccines enhancing GVL reaction and in vitro as a load to antigen presenting cells stimulating reactivity of cytotoxic T-cells [53]. HA-1 and HA-2 are the most intensively explored MiHAs in immunotherapy [12, 52–54].

The aim of this study was to determine MiHA alleles and genotypes enabling to detect their immunogenic disparities in sibling donor-recipient pairs and to explore their influence on the results of allo-HCT.

## 2. Material and Methods

### 2.1. Patients and Donors

62 patients: 34 women and 28 men of median age 38 (range 14–59) years, who received allo-HCT from siblings in the Department of Hematology and Bone Marrow Transplantation, Medical University of Silesia, Katowice, Poland, in years 2000–2008, entered the study. The indication for transplantation was acute myeloid leukemia (45 pts), acute lymphoblastic leukemia (14 pts), chronic myeloid leukemia in chronic phase, myelodysplastic syndrome, and resistant non-Hodgkin's lymphoma (1 pt each). Donors were 30 women and 32 men of median age 35 (range 14–60) years. Median followup was 3 (0.04–10) years.

### 2.2. Transplantation Procedure

Conditioning treatment was myeloablative (CyTBI: cyclophosphamide + total body irradiation in 12 pts, BuCy: busulfan + cyclophosphamide in 33 pts), reduced intensity (TreoFlu: treosulfan + fludarabine in 2 pts, TreoCy: treosulfan + cyclophosphamide in 2 pts), or nonmyeloablative (BuFlu: busulfan + fludarabine in 2 pts). Cumulative doses of drugs used in conditioning were busulfan 16 or 8 mg/kg p.o., cyclophosphamide 120 mg/kg i.v., treosulfan 42 g/m^2^ i.v., fludarabine 150 mg/m^2^ i.v. TBI dose was 12 Gy. Bone marrow was the source of hematopoietic cells in 40 patients, G-CSF-stimulated peripheral blood in 10 and both (harvest of insufficient number of CD34+ cells from the bone marrow followed by peripheral collection) in 12 patients. Details of transplanted cells are presented in Table 3. Standard GVHD prophylaxis consisted of cyclosporine A in initial dose 3 mg/kg i.v. starting from day −1 with dose adjusted to its serum level and shifted to oral administration about day +20, methotrexate 15 mg/m^2^ i.v. on day +1 and 10 mg/m^2^ i.v. on days +3 and +6. Methylprednisolone at dose 2 mg/kg i.v. was the first line therapy of aGVHD symptoms. The criteria defined by Glucksberg were used for the grading of aGVHD; the diagnosis and severity of cGVHD were determined according to NIH (National Institutes of Health) criteria established in 2005 [55].

### 2.3. Methods

DNA of patients and siblings was isolated from peripheral blood in the Biomolecular Laboratory of the Department of Hematology and BMT, Medical University of Silesia. Alleles of 11 autosomal and Y-chromosome encoded MiHAs were analyzed with PCR-SSP method for each donor-recipient pair in the Immunogenetics and HLA Laboratory of the Regional Blood Center in Katowice with the use of Dynal AllSet+ Minor Histocompatibility Antigen Typing Kit, according to a methodology recommended by Leiden University Medical Center. Products obtained in PCR-SSP reaction were analyzed on agarose gel and each detected allele encoding MiHA was translated into a specific letter code. dbMinor database of LUMC was used to determine the number, direction, and tissue distribution of MiHA mismatches on the base of MiHA alleles and HLA antigens of respective donor-recipient pairs. The study has been approved by the responsible Ethical Committee of Medical University of Silesia.

### 2.4. Statistical Methods

Median, minimal, and maximum values were used to show numeric parameters of donor-recipient groups. Statistical analysis of MiHA mismatches' impact on transplantation outcomes was conducted in accordance to recommendation of EBMT [56]. MiHA mismatches were grouped according to mismatch direction (GVH or HVG), tissue distribution (restricted or broad), and the way of coding (autosomal or by Y-chromosome) in search for their influence on transplant results. Analysed endpoints included overall survival (OS), disease-free survival (DFS), aGVHD, and limited and extensive cGVHD. Kaplan-Meier method was used to estimate the probability of impact of MiHA mismatches on overall survival and disease-free survival. Results were presented as percent ±95% confidence interval (CI). The cumulative incidence method was used to evaluate the probability of relapse and GVHD (acute or chronic) in order to account events which may influence the outcome as a competing risk. Results were presented also in percent ±95% CI. Results with significance level *P* < 0.05 were considered statistically significant.

## 3. Results

### 3.1. Occurrence of Alleles and Genotypes and Their Mismatches

Immunogenic MiHA mismatches were detected in 42 (68%) donor-recipient pairs; 20 (32%) pairs had no mismatched MiHAs. Unidirectional HVG-directed disparities were observed in 18 (29%) pairs (in 9 pairs MiHA mismatches were encoded by Y-chromosome, in 8 pairs autosomally, and in 1 pair both autosomally and by Y-chromosome) and GVH-directed MiHA disparities were observed in another 18 (29%) pairs (in 9 pairs MiHA mismatches were Y-chromosome encoded, in 7 pairs autosomally, and in 2 pairs both autosomally and Y-chromosome encoded). In 6 (10%) pairs bi-directional (both HVG and GVH in the same donor-recipient pairs) MiHA mismatches were observed. The direction of MiHA mismatches is presented in Table 4 and the distribution of 11 MiHA alleles and genotypes in 62 related donor-recipient pairs is presented in Tables 5 and 6.

### 3.2. Impact of Immunogenic MiHA Mismatches on Allo-HCT Outcomes

**Figure 1:**
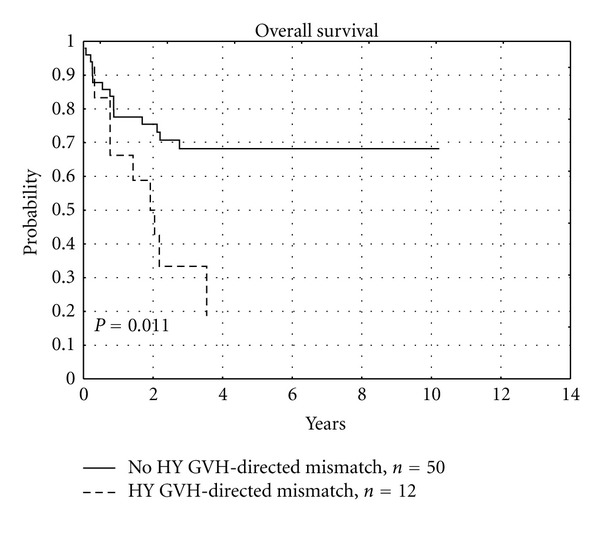
Influence of Y-chromosome encoded GVH-directed MiHA mismatch on overall survival.

**Figure 2:**
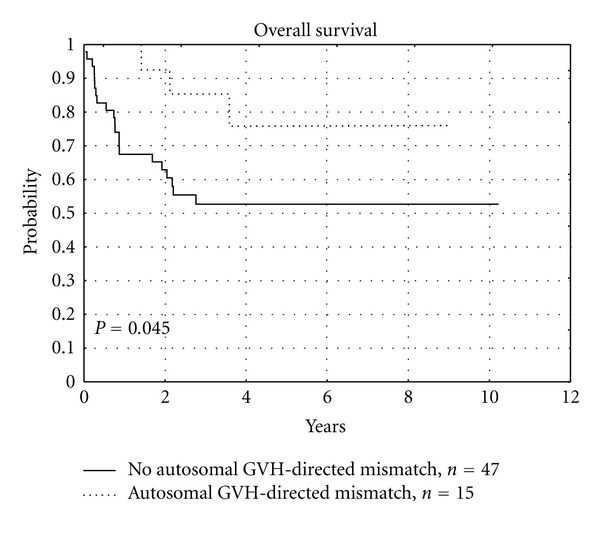
Influence of autosomal GVH-directed MiHA mismatch on overall survival.

Analysis of overall survival showed unfavorable impact of GVH-directed Y-chromosome encoded MiHA mismatches (*P* = 0.011), as presented in Figure 1 and Table 7, and favorable trend in case of GVH-directed autosomal MiHA disparities (*P* = 0.045), as presented in Figure 2 and Table 7.

**Figure 3:**
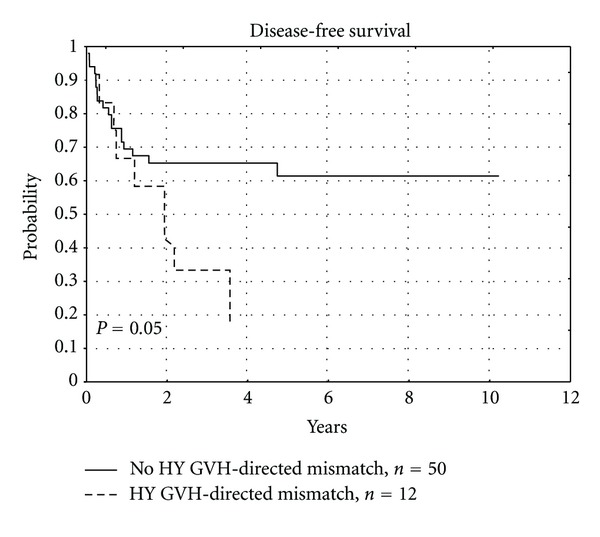
Influence of Y-chromosome encoded GVH-directed MiHA mismatch on disease-free survival.

GVH-directed mismatches of Y-chromosome encoded MiHA influenced unfavorable the disease free-survival (*P* = 0.05), as shown in Figure 3 and Table 7.

**Figure 4:**
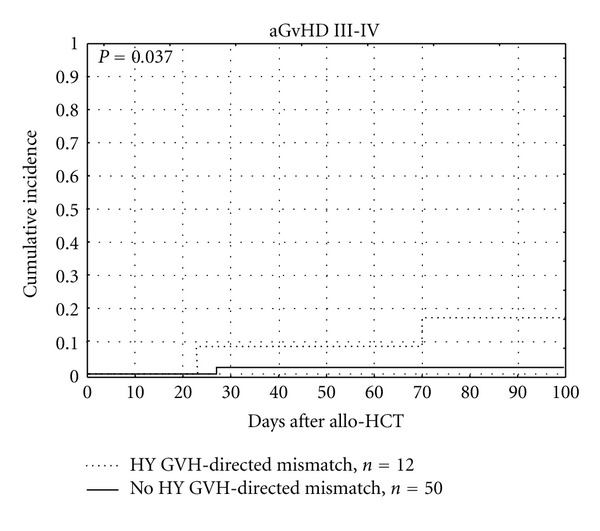
Influence of Y-chromosome encoded GVH-directed MiHA mismatches on serious aGVHD.

Serious (grade III or IV) acute GVHD was observed in 24 patients and it was influenced by Y-chromosome encoded GVH-directed MiHA mismatches (*P* = 0.037), which is presented in Figure 4 and Table 7.

**Figure 5:**
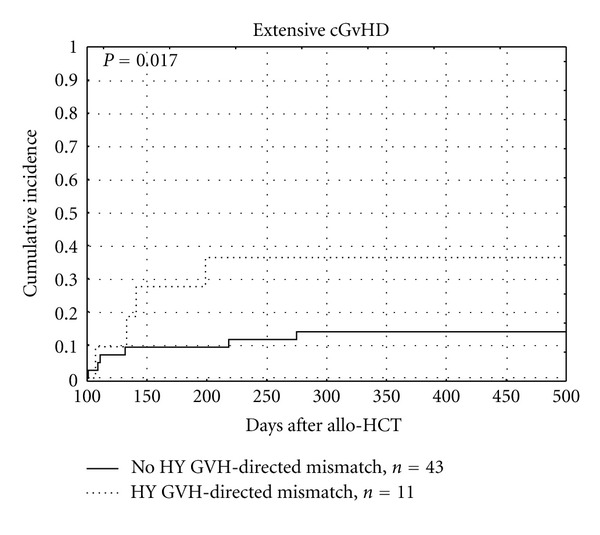
Influence of Y-chromosome encoded GVH-directed MiHA mismatches on extensive cGVHD.

The tissue distribution of GVH- or HVG-directed MiHA mismatches did not influence the incidence of aGVHD, neither grades I-IV, nor II-IV. Higher probability of extensive chronic GVHD was observed when Y-chromosome encoded GVH-directed MiHA mismatches were present (*P* = 0.017, as shown in Figure 5 and Table 7).

The relapse following allo-HCT was observed in 15(24.2%) patients. Lower risk of relapse was observed in patients with HVG-directed MiHA mismatches: both autosomal (0.28(0.18–0.44) versus 0(0-0), *P* = 0.032) and with “restricted” pattern of tissue distribution (0.29(0.18–0.45) versus 0(0-0), *P* = 0.028). These data are presented in Table 7.

## 4. Discussion

Minor histocompatibility antigens belong to genetic factors which may vary between the donor and the recipient despite identical HLA and thus they may influence allo-HCT results. Knowledge of MiHA alleles and genotypes enables to detect their disparities, which could be helpful not only in optimal matching of a donor/recipient pair and in understanding transplant results, but also it may create a chance to the use of MiHA in immunotherapy aiming to improve patients' survival [52–54]. The largest meta-analysis of MiHA distribution was performed by Spierings et al. who described the results of a multicenter trial of 10 MiHA distribution in 5 different ethnic groups worldwide. The study revealed significant differences in the frequency of MiHA alleles in dependence of geographical location, with UGT2B17 being the most variable MiHA [57]. Two MiHA trials have been performed in Polish population till now: in the first one HA-1 was analyzed in a group of 30 sibling pairs [58], another trial concerned the group of 92 unrelated pairs [12]. In our current study alleles and genotypes of 11 MiHAs have been estimated in 62 sibling donor-recipient pairs. Basing on our results and several other studies estimating the occurrence of specific MiHA mismatches in allo-HCT [59, 60], HA-1 can be regarded as a candidate target for immunotherapeutic applications.

We have observed the unfavorable impact of GVH-directed mismatches of Y-chromosome encoded MiHAs on OS (*P* = 0.011) and DFS (*P* = 0.05). Y-chromosome encoded MiHA represents MiHA with “broad” tissue distribution. Attack of donor's T-lymphocytes on recipients' tissues precipitated by HY mismatch could explain the increased occurrence of severe forms of acute and chronic GVHD, leading to earlier deaths of recipients. In our study recipients of allo-HCT from siblings did not receive anti-thymocyte globulin, what probably influenced the worse course, including the fatal course of their GVHD. We have shown that GVH-directed mismatches of HY correlate significantly with serious (III or IV) aGVHD and extensive cGVHD. These results correspond to the reported influence of sex difference on transplant outcomes, especially in the case of female donor to male recipient (FDMR) transplants [61, 62]. Oppositely, Markiewicz et al. in a study of 92 unrelated donor-recipient pairs found that HY mismatches in GVH direction influenced more favorable GVL effect than unfavorable GVHD, what resulted in the increased DFS (*P* = 0.05) [12, 63]. The probable explanation of this difference in MiHAs impact on OS and DFS between related and unrelated allo-HCT may be the use of stronger standard immunosuppressive prophylaxis including pretransplant antithymocyte globulin in unrelated allo-HCT setting. Increased incidence of serious acute and extensive chronic GVHD associated with mismatches of Y-chromosome encoded MiHAs, leading to a worse overall survival, may justify the administration of anti-thymocyte globuline before allo-HCT from sibling female donor to male recipient. Such approach could probably reduce the risk of GVHD originating from GVH-directed HY mismatch.

The analysis of GVH-directed mismatches of autosomal MiHAs, oppositely to HY, showed favorable trend to increase the OS, which was 76% in a mismatched versus 53% in a compatible groups at a 4-year posttransplant. Unlike GVH-directed HY disparities, those of autosomal MiHAs did not increase the occurrence of serious GVHD in our study, which contributed to the better survival. There are reports describing the role of autosomal MiHAs in GVHD: for example, higher risk of aGVHD in the case of autosomal HA-1 incompatibility was reported in Tunisian group of 60 sibling donor-recipient pairs [64]. Others described increased incidence of cGVHD in the case of mismatched autosomal MiHAs localized on hematopoietic cells: HA-1, HA-2, and HA-8 [15, 16, 23, 65]. There are also reports that report no impact of autosomal MiHAs on GVHD [49, 66]. 

One could expect that disparities of MiHAs with broad tissue distribution present in the host should precipitate the posttransplant reaction of donor's lymphocytes and induce the GVHD. Unexpectedly, the tissue distribution of neither GVH- nor HVG-directed MiHA mismatches did not influence the incidence of GVHD. 

Much lower probability of relapse following allo-HCT was observed by us in patients with HVG-, but not with GVH-directed MiHA mismatches. This finding, although intriguing, needs further confirmation as we do not find a reasonable explanation for this result. Japanese group found that GVH-directed HA-1 mismatches were associated with lower risk of relapse [51]. Similarly, experience of Polish group studying MiHAs in unrelated allo-HCT showed seldom episodes of relapse occurring when GVH-directed HY mismatches were present [63].

Results of our study help to explain why posttransplant courses of allo-HCT from siblings may vary despite complete high-resolution HLA-match and why cells interactions between the donor and the recipient may lead to serious complications.

## 5. Conclusions

GVH-directed HY mismatch significantly increased the occurrence of serious acute GVHD and extensive chronic GVHD and finally caused decreased overall survival. GVH-directed mismatches of autosomally encoded MiHAs had no negative effect on overall survival, which in fact was even longer. Findings of our study help to explain why the occurrence of immunological complications and in consequence final results of allo-HCT from high-resolution HLA-matched sibling donors are variable.

## Figures and Tables

**Table 1:** Autosomally encoded MiHA.

MiHA	Restriction	Identification	Clinical trials	Protein	Tissue distribution	Presence on cells
HA-1	HLA-A*02	Den Haan et al. 1998 [14]	Goulmy et al. 1996 [15] Tseng et al. 1999 [16] Gallardo et al. 2001 [17]	HA-1	Restricted	Hematopoietic cells Bronchial carcinomas Cervix carcinoma Breast carcinoma Prostate carcinoma

HA-1/B60	HLA-B*60	Mommaas et al. 2002 [18]	—	HA-1	Restricted	Hematopoietic cells

HA-2	HLA-A*02	Den Haan et al. 1995 [19]	Goulmy et al. 1996 [15]	Myosin 1G	Restricted	Hematopoietic cells

HA-3	HLA-A*01	Spierings et al. 2003 [20]	Tseng et al. 1999 [16]	Lymphoid blast crisis oncogene	Broad	Hematopoietic cells Keratinocytes Fibroblasts PTECs HUVECs Melanocytes

HA-8	HLA-A*02	Brickner et al. 2001 [21]	Akatsuka et al. 2003 [22] Pérez-García et al. 2005 [23]	KIAA0020	Broad	Hematopoietic cells Fibroblasts

HB-1^H/Y^	HLA-B*44	Dolstra et al. 1999 [24]	—	Unknown	Restricted	B cell ALL, EBV-BLCLs

ACC-1	HLA-A*24	Akatsuka et al. 2003 [25]	Nishida et al. 2004 [26]	BCL2A1	Restricted	Hematopoietic cells

ACC-2	HLA-B*44	Akatsuka et al. 2003 [25]	—	BCL2A1	Restricted	Hematopoietic cells

SP110 (HwA-9)	HLA-A*03	Warren et al. 2006 [13]	—	SP110 intranuclear protein	Restricted	Hematopoietic cells IFN—gamma inducible

PANE1 (HwA-10)	HLA-A*03	Brickner et al. 2006 [27]	—	PANE1	Restricted	Lymphoid cells

UGT2B17/A29	HLA-A*29	Murata et al. 2003 [28]	—	UGT2B17	Restricted	Dendritic cells, B-cells, EBV-BLCLs

UGT2B17/B44	HLA-B*44	Terrakura et al. 2007 [29]		UGT2B17	Restricted	Dendritic cells, B-cells, EBV-BLCLs

**Table 2:** Y-chromosome encoded MiHA.

MiHA	Restriction	Identification	Clinical trials	Protein	Tissue distribution	Presence on cells
A1/HY	HLA-A*01	Pierce et al. 1999 [30]	—	USP9Y	Broad	Hematopoietic cells, fibroblasts

A2/HY	HLA-A*02	Meadows et al. 1997 [31]	Goulmy et al. 1996 [15]	SMCY	Broad	Hematopoietic cells, fibroblasts

A33/HY	HLA-A*33	Torikai et al. 2004 [32]	—	TMSB4Y	Broad	Hematopoietic cells

B7/HY	HLA-B*07	Wang et al. 1995 [33]	—	KDMSD	Broad	Hematopoietic cells

B8/HY	HLA-B*08	Warren et al. 2000 [34]	—	UTY	Restricted	Hematopoietic cells

B52/HY	HLA-B*52	Ivanov et al. 2005 [35]	—	RPS4Y1	Restricted	Leukocytes, PHA blasts, EBV-BLCLs, B cells, breast carcinoma, hepatocellular carcinoma, colon adenocarcinoma, AML, ALL multiple myeloma

B60/HY	HLA-B*60	Vogt et al. 2000 [36]	—	UTY	Broad	Hematopoietic cells, fibroblasts

DRB1*1501/HY	HLA-DRB1*15	Zorn et al. 2004 [37]	—	DDX3Y (DBY)	Broad	Hematopoietic cells, fibroblasts

DRB3*0301/HY	HLA-DRB3*0301	Spierings et al.2003 [38]	—	RPS4Y1	Broad	Hematopoietic cells, fibroblasts

DQ5/HY	HLA-DQB1*05	Vogt et al. 2002 [39]	—	DDX3Y (DBY)	Broad	Hematopoietic cells, fibroblasts

Abbreviations: HUVE: human umbilical vein epithelium, PTE: proximal tubular epithelium, EBV-BLCL: Epstein Barr virus transformed B-lymphoblastoid cell lines, and PHA: phytohemagglutinine.

Data in Tables 1 and 2 are based on dbMinor database and materials presented during Minor Histocompatibility Workshop 2005, Leiden University Medical Center; Eric Spierings: minor H antigens: targets for tumor therapy—lecture at the conference “Immunogenetics in hematology and stem cell transplantation”, Wroclaw 09.02.2006 and [8].

**Table 3:** Patients characteristics (*n* = 62).

	Median ( range )	Quartiles
Age (years)		
Donor	35 (14–60)	26–49
Recipient	38 (14–59)	28–47
Time from diagnosis to allo-HCT (years)	0.62 (0.24–12.91)	0.5–1.12

	*n*	%

Sex		
Donor		
Male	32	51.6
Female	30	48.4
Recipient		
Male	28	45.2
Female	34	54.8
Donor/recipient		
Male/male	16	25.8
Female/female	18	29
Male/female	16	25.8
Female/male	12	19.4
Compatibility of ABO blood groups		
Compatible	36	58.1
Minor incompatibility	8	12.9
Major incompatibility	14	22.5
Minor and major incompatibility	4	6.5
Diagnosis		
AML	45	72.5
ALL	14	22.5
CML	1	1.61
MDS	1	1.61
NHL	1	1.61
Regimen		
TBI + cyclophosphamide	12	19.35
Chemotherapy		
Busulfan + cyclophosphamide	33	53.2
Treosulfan + fludarabine	13	20.96
Busulfan + fludarabine	2	3.22
Treosulfan + cyclophosphamide	2	3.22
Source of hematopoietic cells		
Bone marrow	40	64.5
Peripheral blood	10	16.1
Bone marrow and peripheral blood	12	19.4

	Median (range)	Quartiles

Number of transplanted cells		
Nucleated cells (NC) × 10*e*8/kg	3.51 (0.12–72.15)	2.34–5.84
CD34(+) × 10*e*6/kg	2.77 (0.95–10.50)	1.68–4.19
CD3(+) × 10*e*7/kg	3.84 (0.20–46.90)	2.71–18.01
Time range of allo-HCT	01.2000–12.2008	

**Table 4:** The occurrence of MiHA mismatches in GVH and HVG direction in 62 related donor-recipient pairs.

Immunogenic MiHA mismatches	In GVH direction	
	Present	Absent
In HVG direction		
Present	10% (6 pairs)	29% (18 pairs)
Absent	29% (18 pairs)	32% (20 pairs)

**Table 5:** Distribution of 11 MiHA alleles in 62 related donor-recipient pairs.

MiHA	Allele	Recipient	Donor
HA-1	H	38.5%	41.8%
	R	61.5%	58.2%
HA-2	V	78.7%	73.0%
	M	21.3%	27.0%
HA-3	T	68.0%	70.5%
	M	32.0%	29.5%
HA-8	R	45.9%	45.9%
	P	54.1%	54.1%
HB-1	H	62.3%	64.8%
	Y	37.7%	35.2%
ACC-1	Y	23.0%	20.5%
	C	77.0%	79.5%
ACC-2	D	20.5%	19.7%
	G	79.5%	80.3%
SP110 (HwA9)	R	58.2%	58.2%
	G	41.8%	41.8%
PANE1 (HwA10)	R	67.2%	68.9%
	*	32.8%	31.1%
UGT2B17	+	86.9%	90.2%
	−	13.1%	9.8%
HY	+	50.8%	54.1%
	−	49.2%	45.9%

**Table 6:** Distribution of MiHA genotypes' frequencies in 62 related donor-recipient pairs.

MiHA	Genotype	Recipient	Donor
HA-1	HH	13.1%	16.4%
	HR	50.8%	50.8%
	RR	36.1%	32.8%
HA-2	VV	59.0%	50.8%
	VM	39.3%	44.3%
	MM	1.6%	4.9%
HA-3	TT	44.3%	47.5%
	TM	47.5%	45.9%
	MM	8.2%	6.6%
HA-8	RR	27.9%	27.9%
	RP	36.1%	36.1%
	PP	36.1%	36.1%
HB-1	HH	34.4%	36.1%
	HY	55.7%	57.4%
	YY	9.8%	6.6%
ACC-1	YY	4.9%	1.6%
	YC	36.1%	37.7%
	CC	59.0%	60.7%
ACC-2	DD	3.3%	0.0%
	DG	34.4%	39.3%
	GG	62.3%	60.7%
SP110 (HwA9)	RR	27.9%	31.1%
	RG	60.7%	54.1%
	GG	11.5%	14.8%
PANE1 (HwA10)	RR	42.6%	42.6%
	R*	49.2%	52.5%
	**	8.2%	4.9%

++ or +− genotypes' frequencies of UGT2B17 and HY are equal to the frequency of alleles + and their − − genotypes' frequencies are equal to the frequency of alleles − presented in Table 5.

**Table 7:** Influence of MiHA mismatches on allo-HCT outcomes.

Analyzed outcome	Analyzed MiHA	Direction of mismatch	Presence of mismatch	*n*	Probability (95% CI)	*P*
Overall survival	Autosomal	GVH	Yes	15	2 yrs: 0.9286 (0.5278–0.9892)	0.045
					4 yrs: 0.7619 (0.3481–0.9130)	
			No	47	2 yrs: 0.6046 (0.4329–0.7243)	
					4 yrs: 0.5265 (0.3511–0.6545)	
	HY	GVH	Yes	12	2 years: 0.4167 (0.0590–0.6384)	0.011
					3 years: 0.3333 (0.0054–0.5532)	
			No	50	2 years: 0.7546 (0.5986–0.8500)	
					3 years: 0.6822 (0.5152–0.7916)	

Disease-free survival	HY	GVH	Yes	12	2 years: 0.4167 (0.0590–0.6384)	0.050
					3 years: 0.3333 (0.0054–0.5532)	
			No	50	2 years: 0.6526 (0.4896–0.7635)	
					3 years: 0.6526 (0.4896–0.7635)	

Serious aGVHD	HY	GVH	Yes	12	0.1667 (0.0470–0.5906)	0.037
			No	50	0.0200 (0.0029–0.1392)	

Extensive cGVHD	HY	GVH	Yes	11	0.3636 (0.1664–0.7947)	0.017
			No	43	0.1395 (0.0664–0.2931)	

Relapse	Autosomal	HVG	Yes	12	0 (0-0)	0.032
			No	50	0.2836 (0.1818–0.4423)	
	Restricted	HVG	Yes	13	0 (0-0)	0.028
			No	49	0.2879 (0.1849-0.4482)	

## References

[1] Greinix H, Greinix HT (2008). Introduction. *Graft -Versus-Host Disease*.

[2] Szczeklik A, Szczeklik A (2011). Internal Medicine State of the art in 2011. *Practical Medicine*.

[3] Shaw BE, Madrigal A, Apperley J, Carreras E, Gluckman E, Masszi T (2012). Immunogenetics of allogeneic HSCT. *Haematopoietic Stem Cell Transplantation, ESH-EBMT Handbook*.

[4] Counce S, Smith P, Barth R, Snell GD (1956). Strong and weak histocompatibility gene differences in mice and their role in the rejection of homografts of tumors and skin. *Annals of Surgery*.

[5] Snell GD (1964). Methods for study of histocompatibility genes and isoantigens. *Methods in Medical Research*.

[6] Goulmy E (2006). Minor histocompatibility antigens: from transplantation problems to therapy of cancer. *Human Immunology*.

[7] Simpson E, Roopenian D, Goulmy E (1998). Much ado about minor histocompatibility antigens. *Immunology Today*.

[8] Spierings E, Goulmy E (2005). Expanding the immunotherapeutic potential of minor histocompatibility antigens. *Journal of Clinical Investigation*.

[9] Spierings E, Wieles B, Goulmy E (2004). Minor histocompatibility antigens—big in tumour therapy. *Trends in Immunology*.

[10] Mommaas B (2006). *The human minor histcompatibility antigen HA-1: its processing, presentation and recognition [Ph.D. thesis]*.

[11] Falkenburg JHF, van de Corput L, Marijt EWA, Willemze R (2003). Minor histocompatibility antigens in human stem cell transplantation. *Experimental Hematology*.

[12] Markiewicz M (2007). *Influence of donor selection and occurrence and impact of minor histocompatibility antigens' mismatches on results of hematopoietic cells transplantations from HLA-matched unrelated donors [Habilitation thesis]*.

[13] Warren EH, Vigneron NJ, Gavin MA (2006). An antigen produced by splicing of noncontiguous peptides in the reverse order. *Science*.

[14] den Haan JMM, Meadows LM, Wang W (1998). The minor histocompatibility antigen HA-1: a diallelic gene with a single amino acid polymorphism. *Science*.

[15] Goulmy E, Schipper R, Pool J (1996). Mismatches of minor histocompatibility antigens between HLA-identical donors and recipients and the development of graft-versus-host disease after bone marrow transplantation. *The New England Journal of Medicine*.

[16] Tseng LH, Lin MT, Hansen JA (1999). Correlation between disparity for the minor histocompatibility antigen HA-1 and the development of acute graft-versus-host disease after allogeneic marrow transplantation. *Blood*.

[17] Gallardo D, Aróstegui JI, Balas A (2001). Disparity for the minor histocompatibility antigen HA-1 is associated with an increased risk of acute graft-versus-host disease (GvHD) but it does not affect chronic GvHD incidence, disease-free survival or overall survival after allogeneic human leucocyte antigen-identical sibling donor transplantation. *British Journal of Haematology*.

[18] Mommaas B, Kamp J, Drijfhout JW (2002). Identification of a novel HLA-B60-restricted T cell epitope of the minor histocompatibility antigen HA-1 locus. *Journal of Immunology*.

[19] den Haan JMM, Sherman NE, Blokland E (1995). Identification of a graft versus host disease-associated human minor histocompatibility antigen. *Science*.

[20] Spierings E, Brickner AG, Caldwell JA (2003). The minor histocompatibility antigen HA-3 arises from differential proteasome-mediated cleavage of the lymphoid blast crisis (Lbc) oncoprotein. *Blood*.

[21] Brickner AG, Warren EH, Caldwell JA (2001). The immunogenicity of a new human minor histocompatibility antigen results from differential antigen processing. *Journal of Experimental Medicine*.

[22] Akatsuka Y, Warren EH, Gooley TA (2003). Disparity for a newly identified minor histocompatibility antigen, HA-8, correlates with acute graft-versus-host disease after haematopoietic stem cell transplantation from an HLA-identical sibling. *British Journal of Haematology*.

[23] Pérez-García A, de la Cámara R, Torres A, González M, Jiménez A, Gallardo D (2005). Minor histocompatibility antigen HA-8 mismatch and clinical outcome after hla-identical sibling donor allogeneic stem cell transplantation. *Haematologica*.

[24] Dolstra H, Fredrix H, Maas F (1999). A human minor histocompatibility antigen specific for B cell acute lymphoblastic leukemia. *Journal of Experimental Medicine*.

[25] Akatsuka Y, Nishida T, Kondo E (2003). Identification of a polymorphic gene, BCL2A1, encoding two novel hematopoietic lineage-specific minor histocompatibility antigens. *Journal of Experimental Medicine*.

[26] Nishida T, Akatsuka Y, Morishima Y (2004). Clinical relevance of a newly identified HLA-A24-restricted minor histocompatibility antigen epitope derived from BCL2A1, ACC-1, in patients receiving HLA genotypically matched unrelated bone marrow transplant. *British Journal of Haematology*.

[27] Brickner AG, Evans AM, Mito JK (2006). The PANE1 gene encodes a novel human minor histocompatibility antigen that is selectively expressed in B-lymphoid cells and B-CLL. *Blood*.

[28] Murata M, Warren EH, Riddell SR (2003). A human minor histocompatibility antigen resulting from differential expression due to a gene deletion. *Journal of Experimental Medicine*.

[29] Terakura S, Murata M, Warren EH (2007). A single minor histocompatibility antigen encoded by UGT2B17 and presented by human leukocyte antigen-A*2902 and -B*4403. *Transplantation*.

[30] Pierce RA, Field ED, den Haan JMM (1999). Cutting edge: the HLA-A*0101-restricted HY minor histocompatibility antigen originates from DFFRY and contains a cysteinylated cysteine residue as identified by a novel mass spectrometric technique. *Journal of Immunology*.

[31] Meadows L, Wang W, den Haan JMM (1997). The HLA-A*0201-restricted H-Y antigen contains a posttranslationally modified cysteine that significantly affects T cell recognition. *Immunity*.

[32] Torikai H, Akatsuka Y, Miyazaki M (2004). A novel HLA-A*3303-restricted minor histocompatibility antigen encoded by an unconventional open reading frame of human TMSB4Y gene. *Journal of Immunology*.

[33] Wang W, Meadows LR, den Haan JMM (1995). Human H-Y: a male-specific histocompatibility antigen derived from the SMCY protein. *Science*.

[34] Warren EH, Gavin MA, Simpson E (2000). The human UTY gene encodes a novel HLA-B8-restricted H-Y antigen. *Journal of Immunology*.

[35] Ivanov R, Aarts T, Hol S (2005). Identification of a 40S ribosomal protein S4-derived H-Y epitope able to elicit a lymphoblast-specific cytotoxic T lymphocyte response. *Clinical Cancer Research*.

[36] Vogt MHJ, Goulmy E, Kloosterboer FM (2000). UTY gene codes for an HLA-B60-restricted human male-specific minor histocompatibility antigen involved in stem cell graft rejection: characterization of the critical polymorphic amino acid residues for T-cell recognition. *Blood*.

[37] Zorn E, Miklos DB, Floyd BH (2004). Minor histocompatibility antigen DBY elicits a coordinated B and T cell response after allogeneic stem cell transplantation. *Journal of Experimental Medicine*.

[38] Spierings E, Vermeulen CJ, Vogt MH (2003). Identification of HLA class II-restricted H-Y-specific T-helper epitope evoking CD4+ T-helper cells in H-Y-mismatched transplantation. *The Lancet*.

[39] Vogt MHJ, van den Muijsenberg JW, Goulmy E (2002). The DBY gene codes for an HLA-DQ5-restricted human male-specific minor histocompatibility antigen involved in graft-versus-host disease. *Blood*.

[40] http://www.lumc.nl/dbminor.

[41] Klein CA, Wilke M, Pool J (2002). The hematopoietic system-specific minor histocompatibility antigen HA-1 shows aberrant expression in epithelial cancer cells. *Journal of Experimental Medicine*.

[42] Fujii N, Hiraki A, Ikeda K (2002). Expression of minor histocompatibility antigen, HA-1, in solid tumor cells. *Transplantation*.

[43] Spierings E, Drabbels J, Hendriks M (2006). A uniform genomic minor histocompatibility antigen typing methodology and database designed to facilitate clinical applications. *PLoS ONE*.

[44] Simpson E, Scott D, James E (2001). Minor H antigens: genes and peptides. *European Journal of Immunogenetics*.

[45] Laylor R, Cannella L, Simpson E, Dazzi F (2004). Minor histocompatibility antigens and stem cell transplantation. *Vox Sanguinis*.

[46] Spaapen R, Mutis T (2008). Targeting haematopoietic-specific minor histocompatibility antigens to distinguish graft-versus-tumour effects from graft-versus-host disease. *Best Practice and Research*.

[47] Stern M, Brand R, de Witte T (2008). Female-versus-male alloreactivity as a model for minor histocompatibility antigens in hematopoietic stem cell transplantation. *American Journal of Transplantation*.

[48] Markiewicz M, Siekiera U, Dzierzak-Mietla M, Zielinska P, Kyrcz-Krzemien S (2010). The impact of H-Y mismatches on results of HLA-matched unrelated allogeneic hematopoietic stem cell transplantation. *Transplantation Proceedings*.

[49] Lin MT, Gooley T, Hansen JA (2001). Absence of statistically significant correlation between disparity for the minor histocompatibility antigen HA-1 and outcome after allogeneic hematopoietic cell transplantation. *Blood*.

[50] Falkenburg JHF, Goselink HM, van der Harst D (1991). Growth inhibition of clonogenic leukemic precursor cells by minor histocompatibility antigen-specific cytotoxic T lymphocytes. *Journal of Experimental Medicine*.

[51] Katagiri T, Shiobara S, Nakao S (2006). Mismatch of minor histocompatibility antigen contributes to a graft-versus-leukemia effect rather than to acute GVHD, resulting in long-term survival after HLA-identical stem cell transplantation in Japan. *Bone Marrow Transplantation*.

[52] Falkenburg JHF, Willemze R (2004). Minor histocompatibility antigens as targets of cellular immunotherapy in leukaemia. *Best Practice and Research*.

[53] Jedema I, Falkenburg JHF, Apperley J, Carreras E, Gluckman E, Masszi T (2012). Immunotherapy post-transplant. *Haematopoietic Stem Cell Transplantation, ESH-EBMT Handbook*.

[54] Dickinson A, Greinix HT (2008). Biomarkers in acute and chronic GVHD. *Graft -Versus-Host Disease*.

[55] Apperley J, E J, Masszi T, Apperley J, Carreras E, Gluckman E, Masszi T (2012). Graft-versus-host disease. *Haematopoietic Stem Cell Transplantation, ESH-EBMT Handbook*.

[56] Szydlo RM, Apperley J, Carreras E, Gluckman E, Masszi T (2012). The Statistical evaluation of HSCT data. *Haematopoietic Stem Cell Transplantation, ESH-EBMT Handbook*.

[57] Spierings E, Hendriks M, Absi L (2007). Phenotype frequencies of autosomal minor histocompatibility antigens display significant differences among populations. *PLoS Genetics*.

[58] Siekiera U, Janusz J (2001). Human minor histocompatibility antigens (mHag) in HLA-ABC, DR, DQ matched sib-pairs. *Transfusion Clinique et Biologique*.

[59] Lio HY, Tang JL, Wu J, Wu SJ, Lin CY, Yang YC (2010). Minor histocompatibility antigen HA-1 and HA-2 polymorphisms in Taiwan: frequency and application in hematopoietic stem cell transplantation. *Clinical Chemistry and Laboratory Medicine*.

[60] Jung H, Ki CS, Kim JW, Kang ES (2012). Frequencies of 10 autosomal minor histocompatibility antigens in Korean population and estimated disparities in unrelated hematopoietic stem cell transplantation. *Tissue Antigens*.

[61] Randolph SSB, Gooley TA, Warren EH, Appelbaum FR, Riddell SR (2004). Female donors contribute to a selective graft-versus-leukemia effect in male recipients of HLA-matched, related hematopoietic stem cell transplants. *Blood*.

[62] Mutis T, Gillespie G, Schrama E, Falkenburg JHF, Moss P, Goulmy E (1999). Tetrameric HLA class I-minor histocompatibility antigen peptide complexes demonstrate minor histocompatibility antigen-specific cytotoxic T lymphocytes in patients with graft-versus-host disease. *Nature Medicine*.

[63] Markiewicz M, Siekiera U, Karolczyk A (2009). Immunogenic disparities of 11 minor histocompatibility antigens (mHAs) in HLA-matched unrelated allogeneic hematopoietic SCT. *Bone Marrow Transplantation*.

[64] Sellami MH, Torjemane L, de Arias AE (2010). Does minor histocompatibility antigen HA-1 disparity affect the occurrence of graft-versus-host disease in tunisian recipients of hematopoietic stem cells?. *Clinics*.

[65] Socié G, Loiseau P, Tamouza R (2001). Both genetic and clinical factors predict the development of graft-versus-host disease after allogeneic hematopoietic stem cell transplantation. *Transplantation*.

[66] Tait BD, Maddison R, McCluskey J (2001). Clinical relevance of the minor histocompatibility antigen HA-1 in allogeneic bone marrow transplantation between HLA identical siblings. *Transplantation Proceedings*.

